# Semantic Point Cloud Mapping of LiDAR Based on Probabilistic Uncertainty Modeling for Autonomous Driving

**DOI:** 10.3390/s20205900

**Published:** 2020-10-19

**Authors:** Sungjin Cho, Chansoo Kim, Jaehyun Park, Myoungho Sunwoo, Kichun Jo

**Affiliations:** 1 Department of Automotive Engineering, Hanyang University, Seoul 04763, Korea; sungjincho215@gmail.com (S.C.); chansoo7857@gmail.com (C.K.); chang8224@gmail.com (J.P.); msunwoo@hanyang.ac.kr (M.S.); 2 Department of Smart Vehicle Engineering, Konkuk University, Seoul 05029, Korea

**Keywords:** semantic point cloud mapping, uncertainty probability, LiDAR, deep learning-based semantic segmentation, graph SLAM, autonomous vehicle

## Abstract

LiDAR-based Simultaneous Localization And Mapping (SLAM), which provides environmental information for autonomous vehicles by map building, is a major challenge for autonomous driving. In addition, the semantic information has been used for the LiDAR-based SLAM with the advent of deep neural network-based semantic segmentation algorithms. The semantic segmented point clouds provide a much greater range of functionality for autonomous vehicles than geometry alone, which can play an important role in the mapping step. However, due to the uncertainty of the semantic segmentation algorithms, the semantic segmented point clouds have limitations in being directly used for SLAM. In order to solve the limitations, this paper proposes a semantic segmentation-based LiDAR SLAM system considering the uncertainty of the semantic segmentation algorithms. The uncertainty is explicitly modeled by proposed probability models which are come from the data-driven approaches. Based on the probability models, this paper proposes semantic registration which calculates the transformation relationship of consecutive point clouds using semantic information with proposed probability models. Furthermore, the proposed probability models are used to determine the semantic class of the points when the multiple scans indicate different classes due to the uncertainty. The proposed framework is verified and evaluated by the KITTI dataset and outdoor environments. The experiment results show that the proposed semantic mapping framework reduces the errors of the mapping poses and eliminates the ambiguity of the semantic information of the generated semantic map.

## 1. Introduction

Simultaneous Localization and Mapping (SLAM), one of the main challenges for autonomous driving technology, involves a system that simultaneously performs the localization of the vehicle itself and the map building without any prior knowledge [1]. In order to achieve this purpose, SLAM relies on various types of sensors to acquire environmental information. Among the perception sensors, Light Detection And Ranging (LiDAR) sensor is widely used because it does not suffer from the failures caused by illumination changes and can provide high-resolution point cloud for the surrounding environments. The point clouds mainly describe the geometric information of the surroundings without semantic information. As a consequence, the map from the LiDAR-based SLAM only contains the geometry information of the environments [2].

Recently, in addition to the geometry information, LiDAR point clouds can provide the semantic information owing to the success of deep neural network in point classification tasks [3]. Many deep learning-based methods have been proposed to classify the point cloud into predefined semantic classes such as buildings, vehicles, trees, and poles using various deep neural network-based approaches. Moreover, these methods demonstrated success in their online segmentation performance.

Under this background, the deep learning-based semantic segmentation methods have been recently adopted for the LiDAR-based SLAM problem. There are several advantages to the combination of these two technologies. First of all, the inclusion of semantic information within an environmental map provides a much greater range of functionality for autonomous vehicles than geometry alone [4]. For example, the semantic information in the map allows the autonomous vehicles to know what objects are around them even when the sensors cannot detect the objects, which give a cue for the drivable region. Secondly, semantic information is also useful for the mapping process. The point cloud registration, key technology for LiDAR-based SLAM, estimates the relative transformation between two consecutive poses by matching the point clouds from the poses [5]. If the environments only consist of static objects, the point cloud registration can provide an accurate transformation result. However, in real driving environments, there are a lot of dynamic objects which cause negative effects by moving during data acquisition [6]. In this situation, the semantic segmentation methods help to distinguish the dynamic objects because they provide the class of each point such as a car, pedestrian, and so on [7]. Another advantage of the semantic segmentation for the SLAM is that it can make the corresponding pairs for the point cloud registration. When the consecutive point clouds are segmented with their semantic information, the point cloud registration can be performed between the points classified as the same class, which improves the registration performance.

**Figure 1:**
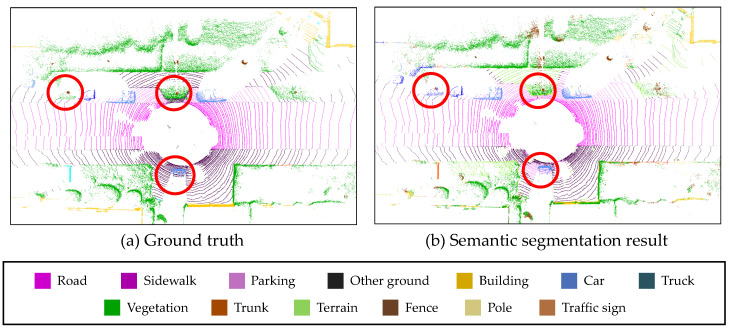
Example of the uncertainty of the semantic segmentation.

However, there are some considerations for addressing the semantic segmentation for the SLAM algorithm. Although the semantic segmentation algorithms provide the semantic information of the surrounding points, the dynamic objects cannot be clearly distinguished only using the semantic information. The points segmented by car have the potential to move, however, they are not always dynamic objects. For example, under specific situations such as parking lot, the parked vehicles may help the point cloud registration as the static objects. Besides, a more important factor is that semantic segmentation results always contain their classification uncertainty caused by the characteristics of the segmentation algorithms as shown in Figure 1. In this figure, RangeNet++ [8] is used for semantic segmentation. Even though RangeNet++ is one of the state-of-the-art algorithms for semantic segmentation, the classification success rate cannot be 100 percent. As previously mentioned, when the point clouds have their own semantic information, the registration performance can be improved by matching the points with the same class. However, due to the classification uncertainty, even though two matching points from consecutive scans are classified as the same class, they cannot be directly used for the registration pair, and vice versa, because the true classes of the points are unknown. Moreover, due to this uncertainty, the same point in multiple-scans from different time steps can represent different semantic information. In that case, the class of the point cannot be determined, which causes the semantic ambiguity of the semantic map.

In this paper, we propose a semantic segmentation-based LiDAR SLAM system considering the uncertainty of the semantic segmentation. The state-of-the-art deep learning-based semantic segmentation algorithms are used for the per-frame point cloud classification. The extracted semantic information is fed into the semantic registration module. In order to reflect the uncertainty of the segmentation algorithms, this paper presents the probability model-based point cloud registration for semantic mapping. In the proposed semantic registration algorithm, two probability models are presented. One is for considering the *movability* of each class, and the other is for the classification performance. For more detail, the probability for the classification performance can describe the confidence of the registration pairs of each class. With the proposed probability model-based point cloud registration algorithm, the ambiguity of both distinction of the dynamic objects and the classification results can be considered. Using the result of the semantic registration, Graph SLAM provides the vehicle poses by optimizing the graph structure which consists of nodes (vehicle pose) and edges (the geometric relationship between the nodes). After the pose optimization, semantic segmented point cloud from each pose should be accumulated for the semantic map generation. The multiple scans can indicate different classes for one point due to the uncertainty. In order to determine the class, this paper proposes the probability-based point cloud accumulation method. During the accumulation process, the semantic information of each point is determined by the proposed probability model for the semantic map. The proposed algorithm is evaluated with both the open benchmark datasets (KITTI [9]) and large-scale datasets from real driving environments.

The main contributions of this paper are summarized as follows:

1. This paper proposes the probability model-based point cloud registration method in order to consider the two types of uncertainty; movement probability of each class and classification probability of the semantic segmentation algorithms.
2. This paper proposes the probability-based point cloud accumulation method in order to determine the semantic information of the semantic map.

The remainder of the paper is organized as follows. Section 2 is about related previous studies and Section 3 presents the system architecture of the proposed mapping system. Section 4, Section 5 and Section 6 describe the detailed methods of the proposed semantic mapping system considering the uncertainty. Section 7 provides the experimental results and Section 8 is for the conclusion.

## 2. Related Work

### 2.1. LiDAR-Based SLAM

LiDAR-based SLAM problems have been researched by thousands of engineers. As a starting point of the LiDAR-based SLAM, a lot of point cloud registration methods were introduced [10,11,12]. These methods provide the transformation relationship between two point clouds, which can be used for LiDAR mapping. For the SLAM problem, the Graph SLAM is one of the representative methods [13]. Graph SLAM optimizes the vehicle poses using point cloud registration as an optimization constraint [14]. In [15], Graph SLAM was adopted for the grid mapping based on an evidential theory. Alternatively, Zhang et al. proposed a low-drift and online LiDAR-only mapping method [16,17], and [18] introduced LeGO-LOAM using geometric feature-based LiDAR odometry estimation for a low-power embedded system. Moreover, the authors of [19] improved the LOAM using a geometric feature-based loop closure method. They performed the loop closure matching by the extracted geometric features such as planar features and edge features from the point cloud. Especially, Li et al. [20] and Liu et al. [21] focused on the ground plane for robust LiDAR odometry estimation. They extracted the points from the ground plane and used the points as new constraints for mapping. Besides, [22] proposed SROM which separated the registration into two steps; Phase Only Correlation (POC)-based rough transformation and point to plane ICP-based fine transformation. For the off-road environments, the authors of [23] provided comparison results of the point cloud registration methods for LiDAR-based mapping. These previous methods only used the geometric information of the point cloud, not the semantic information.

### 2.2. Point Cloud Semantic Segmentation

Many deep learning-based methods for semantic segmentation of point cloud have been proposed recently. RangeNet++ [8], a representative method of Convolutional Neural Network (CNN)-based semantic segmentation, classifies the point cloud using spherical projection. After the classification in 2-D projection, the results are back-projected to the 3-D point cloud. RangeNet++ used Darknet [24], which showed the successful performance in the 2D image semantic segmentation task, as a backbone and proposed a GPU-enabled KNN algorithm to smooth the labels from the Darknet backbone. As another CNN-based method, SqueezeSeg [25] proposed the fire module which applied the 1 × 1 convolution and 3 × 3 convolution in parallel to reduce the size of the CNN model and its computations. Furthermore, to improve the segmentation performance in boundary points, the model refined the labels using Conditional Random Field. SqueezeSegV2 [26] added some residual blocks called the Contextual Aggregation Module in the feature extraction layer of the SqueezeSeg model, so the model demonstrated the robust segmentation in a noisy point cloud. 3-D CNN based methods, which keep the original dimension of the point cloud, were also introduced in [27,28]. These methods voxelized the point cloud and applied the 3-D CNN for semantic segmentation. Furthermore, Graham et al. [29] proposed a submanifold sparse convolutional network to solve the computation time issues. Alternatively, point-wise semantic segmentation methods have been researched. PointNet [3], a representative method of point-wise semantic segmentation, proposed a novel type of neural network that directly uses point clouds. The method was evaluated with unordered and irregular point cloud datasets. Based on the PointNet, PointNet++ [30] adopted hierarchical features with increasing scales of contexts. Moreover, VoteNet developed the PointNet++ using an end-to-end object detection network [31]. The state-of-the-art methods for point cloud semantic segmentation mentioned above inevitably contain the uncertainty caused by the classification performance.

### 2.3. Semantic Segmentation-Based SLAM

With the advances in deep learning for semantic segmentation, a lot of researches on combining the semantic information and SLAM were proposed. SemanticFusion [32] is one of the early works that combined the two technologies. It incrementally integrates semantic information into a dense 3-D map. Dube et al. proposed SegMap [33] which is segment-based mapping using data-driven descriptors. The proposed descriptor was extracted by a neural network and the descriptor was used for mapping. Vision-based semantic segmentation was also largely adopted for the point cloud mapping. Ref. [34] introduced semantic 3-D mapping by the data association between 3-D map from the point cloud and CNN based-segmented labeled images. Ref. [35] proposed a dynamic objects-free LOAM system with overlapping the segmented images into LiDAR scans. In order to achieve the same purpose, Han et al. combined ORB-SLAM2 and PSPNet segmentation [36]. For semantic mapping with the stand-alone LiDAR point cloud, Zaganidis et al. proposed semantic-assisted point cloud registration for LiDAR mapping [37,38]. The researches performed point cloud registration only between the same class from the semantic segmentation. In [39], Semantic LOAM (SLOAM) was proposed for forest inventory using an end-to-end pipeline for tree diameter estimation. Furthermore, SuMa++ presented semantic ICP and dynamic filtering using the semantic information from RangeNet++ for semantic mapping [7]. These studies showed the technological breakthrough in the semantic mapping field, however, the uncertainty from the semantic segmentation algorithms was not explicitly considered.

## 3. System Architecture

**Figure 2:**
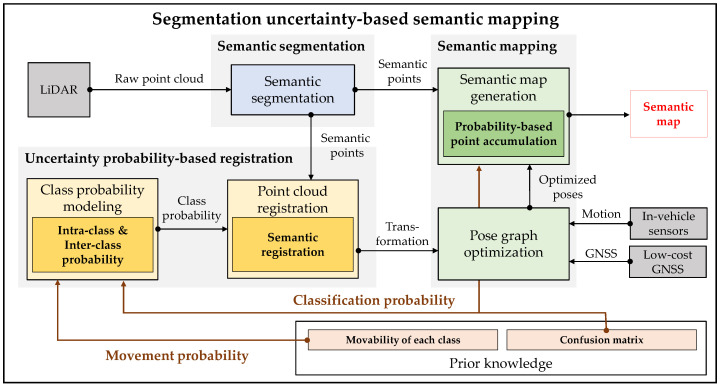
Overall system architecture of the proposed semantic mapping based on probabilistic uncertainty modeling.

The overall system architecture of the proposed semantic mapping consists of three steps as shown in Figure 2. The first step is an online semantic segmentation of the LiDAR point cloud. The previously proposed deep neural network-based semantic segmentation algorithms are used for the segmentation.

The second step is the uncertainty probability-based point cloud registration. Using the prior probabilities, the class probability models which represent the uncertainty are proposed. There are two probability models in the proposed class probability; *Intra-class probability* and *Inter-class probability*. The *Intra-class probability* is for considering the movable objects, which is derived from the movement probability. The *Inter-class probability* is for the registration confidence between the classes, which is come from the classification probability. The modeled class probability is used for point cloud registration. The point cloud registration estimates the transformation relationship between two point clouds. Among the various registration methods, Normal Distribution Transform (NDT) is used in this paper, because it has been proved that NDT registration is robust in real driving environments [40]. In order to reflect the uncertainty into the NDT registration, this paper proposes the semantic registration which combines the class probability and NDT registration. Instead of registration between the same class’ points, the proposed semantic registration algorithm performs the probabilistic matching to resolve the ambiguity from the uncertainty of the semantic segmentation algorithms.

The final step of the proposed mapping framework is semantic mapping. The transformation between two point clouds from the semantic registration is used for the Graph SLAM with additional sensor information such as motion and low-cost GNSS for the pose optimization. In the Graph SLAM, all the poses are represented as nodes. The nodes are optimized based on edge constraints which are generated by the relationship between two nodes such as point cloud registration, motion information, and low-cost GNSS. As a result, the semantic map is generated by accumulating the semantic point clouds using the optimized poses. However, because the semantic information of the segmented point clouds contains uncertainty, the conventional accumulation causes the semantic ambiguity of the generated semantic map. Instead, in the proposed framework, the classification probability is used for the accumulation step. The semantic information of arbitrary point is consistently updated by the proposed probability model which is based on the classification probability.

## 4. Deep Neural Network-Based Semantic Segmentation

**Figure 3:**
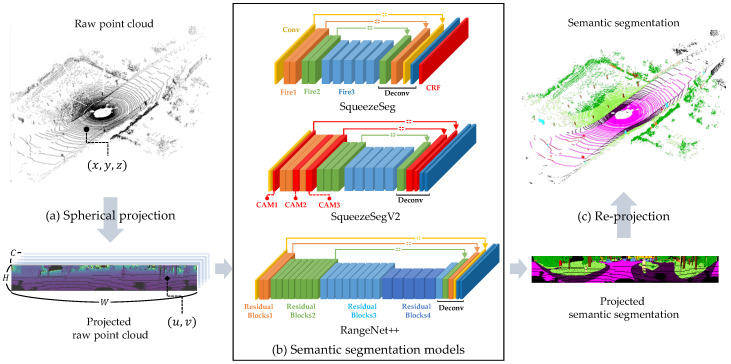
Deep neural network-based semantic segmentation algorithms. (**a**) Spherical projection of the raw point cloud. (**b**) Three semantic segmentation models; SqueezeSeg, SqueezeSegV2, and RangeNet++. (**c**) Re-projection of the segmented image into 3-D point cloud.

The first step of the proposed mapping system is the online semantic segmentation of LiDAR point clouds. The LiDAR point clouds are segmented into semantic classes using the deep neural network-based approaches. This section briefly introduces the deep learning-based point cloud semantic segmentation algorithms. In this paper, three deep neural networks are used; SqueezeSeg [25], SqueezeSegV2 [26], and RangeNet++ [8]. The reason why these networks are chosen is that they were demonstrated by the SemanticKITTI [41], however, it is not an important topic in this paper, because the main purpose of this paper is to consider the uncertainty of arbitrary semantic segmentation algorithm for semantic mapping. Those models project 3-D point cloud into 2-D image and apply 2-D convolutional neural network for semantic segmentation. For the details of each model, Let *N* is the number of points in the point cloud, (H,W) are the height and width of the 2-D projected image and *C* is the channel of the 2-D projected image as shown in Figure 3a. First, all three models go through the same pre-process called spherical projection. The spherical projection maps each point $p_{i}=\left(x,y,z\right)$ to image coordinate (u,v) via Equation (1).

(1) uv=12[1−arctan(y,x)π−1)]W[1−arcsin(zr−1+fovup)fov−1]H

where *r* is the range of each point $\sqrt{x^{2}+y^{2}+z^{2}}$ and $fov=fov_{up}+fov_{down}$ is the vertical field-of-view of the sensor. As a result of the spherical projection, 3-D point cloud converts to 2-D image (H×W×C). In this paper, we define 5 channels; *x*, *y*, *z*, *intensity* and *range* of the point.

The next step is the semantic segmentation of 2-D projected image with their own backbones as described in Figure 3b. SqueezeSeg model proposes the light-weight backbone based on the fire module. SqueezeSegV2 model designs the noise-robust backbone by adding the Contextual Aggregation Module (CAM) to the backbone of the SqueezeSeg model. RangeNet++ refers to the DarkNet backbone which is the state-of-the-art model in image semantic segmentation task and refined the segmentation result from the DarkNet backbone with GPU-enabled KNN algorithm. Finally, those models obtain 3-D semantic segmentation results by re-projecting the 2-D segmented image into the original 3-D point cloud as shown in Figure 3c.

## 5. Uncertainty Probability-Based Point Cloud Registration

The objective of this paper is to reflect the uncertainty of the semantic segmentation algorithms into the semantic mapping framework. The uncertainty is divided into two categories; (1) movement probability of each class, (2) classification probability of the semantic segmentation algorithms. In order to deduce the uncertainty probability model, this paper defines the prior knowledge based on the pre-trained deep learning models. SemanticKITTI open datasets provide a set of point clouds with their semantic information and movement information. Based on the labeled point clouds, we train the deep semantic segmentation networks and extract the movability of each class and confusion matrix. The movability can indicate the movement probability of each class, and the confusion matrix can represent the classification probability of the semantic segmentation algorithms. Based on the prior knowledge, two probability models are proposed, and the probabilities are combined into the point cloud registration to reflect the uncertainty.

### 5.1. Prior Knowledge Definition

**Figure 4:**
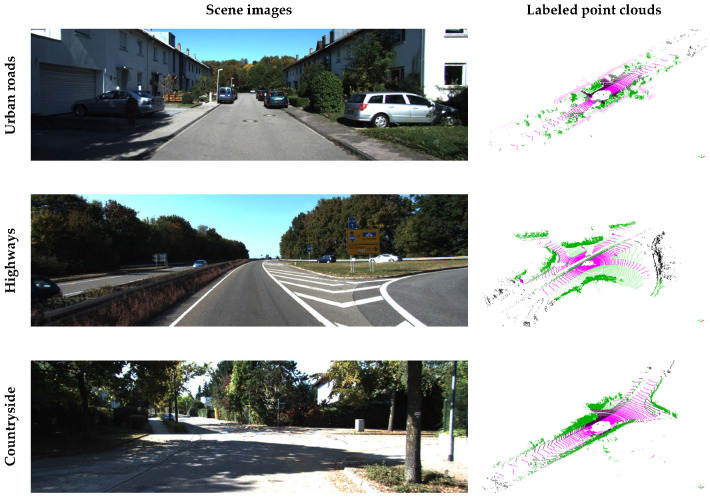
SemanticKITTI dataset for prior knowledge definition.

In order to model the uncertainty of the semantic segmentation algorithms, two types of prior knowledge are defined offline: the movability of each class and confusion matrix. The prior knowledge is calculated using the SemanticKITTI dataset. The SemanticKITTI contains the annotated point cloud into 25 classes collected from 22 different driving environments such as urban roads, highways, and countryside as described in Figure 4. The classes include static objects such as building, pole, and parked car as well as dynamic objects like moving car, person, and so on.

**Figure 5:**
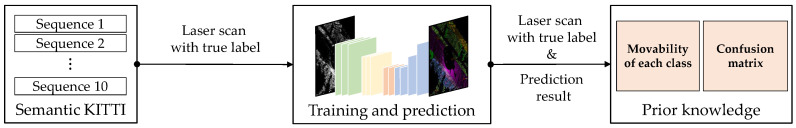
Overall process for the prior knowledge definition.

**Figure 6:**
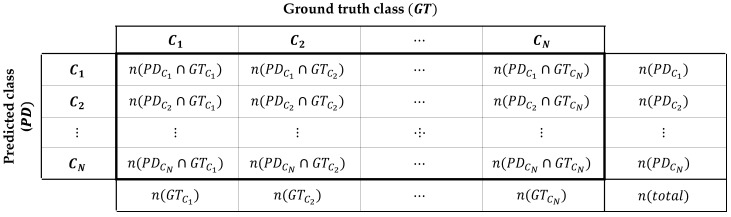
Confusion matrix of the semantic segmentation as a prior knowledge.

The overall process for the prior knowledge definition is shown in Figure 5. The *movability of the class* represents the probability that objects belonging to that class will move. As the SemanticKITTI dataset contains the annotations that the point is moving or not, the movability of the class can be determined by the ratio of the total number of points in the class and the number of moving points in the class. The *confusion matrix* indicates the class-by-class performance of the semantic segmentation of the test scene. Each row of the matrix represents the number of points of a predicted class while each column represents the number of points of a ground truth class in the SemanticKITTI as shown in Figure 6. As same as Section 4, SqueezeSeg, SqueezeSegV2, and RangeNet++ are used for the prior knowledge. In order to obtain the confusion matrix of these networks, three representative environments are selected to cover the various driving environments; urban road, highways, and countryside. When we test the segmentation algorithm in the urban road environment, the model is trained by other datasets in the SemanticKITTI, which is the same for other environments. Finally, three different confusion matrices can be obtained per one segmentation model and those matrices can represent the segmentation performance of the model in overall driving environments.

### 5.2. Class Probability Model

Based on the prior knowledge, two probability models are proposed. The first probability is for the uncertainty about the ambiguity of the movability of the semantic classes. Because moving objects have negative effects on the point cloud registration, they should be eliminated during the mapping process. The semantic segmentation algorithm can help to distinguish the moving points by their semantic classes, however, only the semantic information is not enough to achieve the purpose. In order to model the movability of each class, *Intra-class probability* is proposed in this paper. The intra-class probability is directly modeled by the movement probability from the prior knowledge as described in Equation (2).

(2) $$P\left(C\right)=1-movement\;probability=1-\frac{n_{C}\left(moving\;points\right)}{n_{C}\left(moving\;points\right)+n_{C}\left(nonmoving\;points\right)}$$

As described in Section 5.1, the movability is extracted from the SemanticKITTI dataset which can be divided into three representative environments. Because the movability can be quite different by the environments, the intra-class probability is selectively utilized according to the environments.

The second probability models the uncertainty about the classification performance of the semantic segmentation algorithms. In ideal, if semantic segmentation does not fail to segment the points for all classes, the registration can be performed only between the same class’ points. However, due to inaccuracies in classification, the confidence of the registration pair for points of the same class cannot be determined as 100%. In order to solve the ambiguity from the classification performance, *Inter-class probability* is modeled to assign confidence for registration pairs between classes. Let’s assume that two points in registration pair $\left(p_{i},p_{j}\right)$ are segmented by the class $C_{i}$ and $C_{j}$, however, the true semantic class is unknown. Under the assumption the registration pair should represent the same class, the inter-class probability is defined by the probability that two points have the same true class in this paper. For the point $p_{i}$, the probability which the point has the true class $C_{t}$ can be represented by a conditional probability $p(C_{t}|C_{i})$, and $p(C_{t}|C_{j})$ for the point $p_{j}$. As a result, the inter-class probability between the class $C_{i}$ and $C_{j}$ is modeled by the sum-of-product of the conditional probabilities for all the classes as described in Equation (3).

(3) $$\begin{array}{c} P\left(C_{i},C_{j}\right)=\sum_{t\in \forall C}p\left(C_{t}|C_{i}\right)\cdot p\left(C_{t}|C_{j}\right)\;,where\;p\left(C_{t}|C_{i,j}\right)=\frac{n(PD_{C_{i,j}}\cap GT_{C_{t}})}{n(PD_{C_{i,j}})} \end{array}$$

where C is a set of the semantic classes. Because we defined the conditional probability $p(C_{t}|C_{i})$ as the probability of the class $C_{t}$ when the point is segmented as class $C_{i}$, it is obtained by the confusion matrix (Figure 6) in the form of $\frac{n(PD_{C_{i,j}}\cap GT_{C_{t}})}{n(PD_{C_{i,j}})}$.

In summary, the intra-class probability is the internal probability of unit class which can represent the movability, and the inter-class probability is the cross probability of two classes which can determine the confidence for the registration between the classes.

### 5.3. Semantic Point Cloud Registration

#### 5.3.1. Basic Normal Distribution Transform (NDT)

**Figure 7:**
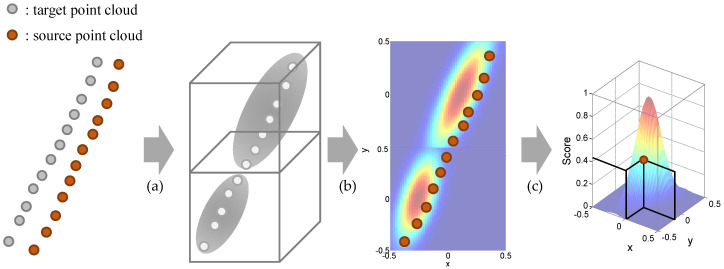
Process of basic Normal Distribution Transform. (**a**) Construction of the normal distribution with target point clouds. (**b**) Projection of the source point clouds into the normal distribution. (**c**) Score calculation for each source point.

In this paper, the Normal Distribution Transform (NDT) is used for the point cloud registration. The NDT is one of the point cloud registration methods which provide the transformation relationship between two consecutive point clouds. The NDT starts by transforming the target point clouds residing within a voxel into a normal distribution as shown in Figure 7a. A mean $q_{k}$ and an covariance matrix $\sum_{k}$ are obtained by the Equation (4).

(4) $$\begin{array}{c} q_{k}=\frac{1}{N_{k}}\sum_{i=0}^{N_{k}-1}x_{k,i}\\ \sum_{k}=\frac{1}{N_{k}}\sum_{i=0}^{N_{k}-1}\left(x_{k,i}-q_{k}\right){\left(x_{k,i}-q_{k}\right)}^{\prime } \end{array}$$

The $x_{k,i}$ represents a position vector $\left(x_{k,i},y_{k,i},z_{k,i}\right)$ for $i^{th}$ point in $k^{th}$ voxel and $N_{k}$ is the number of points in the voxel. With the two parameters, the point cloud in the voxel is modeled by the normal distribution N(q,∑):

(5) $$p_{k}\left(x\right)∼\exp\left(-\frac{\left(x-q_{k}\right)\sum_{k}^{-1}{\left(x-q_{k}\right)}^{\prime }}{2}\right)$$

The purpose of the NDT is to find the optimal transformation matrix p, which maximizes the score for source point clouds $x_{i}$ transformed by the matrix p. The transformed source point clouds $x_{i}^{\prime }$ can be obtained by the transformation relationship $T(x_{i},p)$ as the following equation:

(6) $$x_{i}^{\prime }=T\left(x_{i},p\right)=Rx_{i}+t$$

where R and t is the rotation and translation matrix, respectively. After that, the transformed points are projected into the normal distribution of the corresponding voxels as shown in Figure 7b. With the projected points, the cost function for the NDT is defined as summation of the negative-score functions as shown in Equation (1) and Figure 7c.

(7) $$J\left(p\right)=-\sum_{i}\exp\left(-\frac{\left(x_{i}^{\prime }-q_{i}\right)\sum_{i}^{-1}{\left(x_{i}^{\prime }-q_{i}\right)}^{\prime }}{2}\right)$$

where $q_{i}$ and $\sum_{i}$ are the mean and covariance of the corresponding voxel for the point $x_{i}^{\prime }$. In order to find the optimal 3-D transformation matrix p, Newton’s method is adopted as explained in [42].

#### 5.3.2. Semantic NDT Based on the Class Probability

**Figure 8:**
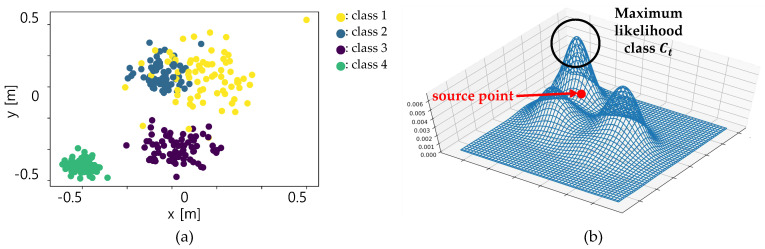
Multi-modal distribution. (**a**) Target point clouds in one voxel (different colors for different classes). (**b**) Multi-modal distribution and maximum likelihood class.

Based on the class probability described in Section 5.2, this subsection provides a detailed method of the semantic NDT. There are two key points of the semantic NDT; Multi-modal normal distribution for the segmented classes using the intra-class probability, Weighted scoring by the inter-class probability. As explained in the previous subsection, the NDT starts with the construction of the normal distribution using the target points in a voxel. Instead of generating a single normal distribution, the multi-modal distribution is constructed per one voxel as shown in Figure 8. The number of distribution is the number of classes which points in the voxel contain. In this point, the intra-class probability is used as the scale factor for combining the multiple distributions into a multi-modal distribution, because the intra-class probability represents the movement probability of each class. The modified mathematical equation for the multi-modal distribution as described in Equations (8) and (9).

(8) $$\begin{array}{c} q_{k,c}=\frac{1}{N_{k,c}}\sum_{i=0}^{N_{k,c}-1}x_{k,c,i}\\ \sum_{k,c}=\frac{1}{N_{k,c}}\sum_{i=0}^{N_{k,c}-1}\left(x_{k,c,i}-q_{k,c}\right){\left(x_{k,c,i}-q_{k,c}\right)}^{\prime } \end{array}$$

(9) $$p_{k}\left(x\right)∼\sum_{c=0}^{N_{c}-1}w_{c}\left(\exp\left(-\frac{\left(x-q_{k,c}\right)\sum_{k,c}^{-1}{\left(x-q_{k,c}\right)}^{\prime }}{2}\right)\right)\;,where\;w_{c}=\eta P\left(C\right)$$

where the additional notation *c* represents the segmented class. P(C) describes the intra-class probability derived by the movement probability of the corresponding class and η is the normalization constant.

The next is the weighted scoring with inter-class probability. The inter-class probability modeled by a cross probability of two classes ($P(C_{1},C_{2})$) represents the registration confidence between classes. In order to reflect the probability into the generated multi-modal distribution, two steps are required. The first step is to determine the maximum likelihood class of the multi-modal normal distribution of each source point. Unlike the basic NDT, in the proposed semantic NDT, one voxel contains multiple normal distributions from the segmented classes. After the projection of the source point into the multi-modal distribution, the maximum likelihood class is determined with the Equation (10).

(10) $$C_{t}=arg\max_{C}p_{k}\left(C|x\right)$$

where $p_{k}\left(C|x\right)$ represents the likelihood of a point x with the corresponding the normal distribution’s class $C_{t}$ in the voxel *k*. As a result, the maximum likelihood class $C_{t}$ means the class of the target distribution of the corresponding source point as described in Figure 8b. After determining the target class $C_{t}$, the inter-class probability is used for the weight factor for the scoring function. The scoring function of the basic NDT (7) is modified by the weighted form as described in Equation (11).

(11) $$J\left(p\right)=-\sum_{i}P\left(C_{i},C_{t}\right)\exp\left(-\frac{\left(x_{i}^{\prime }-q_{i}\right)\sum_{i}^{-1}{\left(x_{i}^{\prime }-q_{i}\right)}^{\prime }}{2}\right)$$

where $C_{i}$ is the class of the $i^{th}$ source point and $C_{t}$ describes the closest target distribution’s class. $P(C_{i},C_{t})$ is the inter-class probability which is obtained in Section 5.2. As a result, the transformation can be obtained by the transformation matrix p.

## 6. Probabilistic Semantic Mapping

The final process of the proposed system is the probability-based semantic map generation. The vehicle poses are firstly optimized by Graph SLAM framework using the transformation from the semantic registration, motion information, and low-cost GNSS. The optimized poses are fed into the semantic map generation with the segmented point clouds. The final semantic map is generated by the probabilistic point cloud accumulation.

### 6.1. Pose Graph Optimization

**Figure 9:**
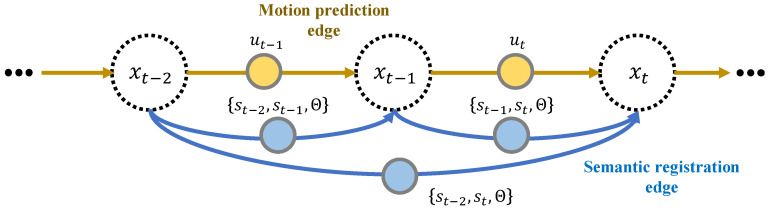
Graphical representation of the pose optimization. The proposed method integrates the probability model-based semantic registration into the pose graph.

In order to optimize the vehicle poses for mapping, Graph SLAM is adopted in this paper. Figure 9 illustrates the graph structure which comprises nodes and edges (relationship between the nodes). In this figure, the white circles of $x_{t-2:t}$ represent the vehicle poses for time steps. The yellow and blue circles indicate the motion information ($u_{t-2:t}$), and segmented point cloud ($s_{t-2:t}$) with class probability (Θ), respectively. With the known information, two types of edges are used for the graph optimization; Motion prediction edge and point cloud registration edge. The motion prediction edge is derived by the vehicle-dynamics-based prediction model, $h_{m}\left(x_{t-1},u_{t}\right)$. The model represents the geometric relationship between the two consecutive pose nodes, $x_{t-1}$ and $x_{t}$, using motion measurement $u_{t}$. When $\epsilon_{u,t}$ is defined as an error of the prediction model caused by the noise of the motion input and the approximation of the prediction model, the motion edge can then be expressed as Equation (12).

(12) $$x_{t}=h_{m}\left(x_{t-1},u_{t}\right)+\epsilon_{m,t}$$

(13) $${\left(x_{t}-h_{m}\left(x_{t-1},u_{t}\right)\right)}^{T}P_{m,t}^{-1}\left(x_{t}-h_{m}\left(x_{t-1},u_{t}\right)\right)$$

Assuming that the prediction error, $\epsilon_{u,t}$, follows the Gaussian distribution with zero mean and covariance $P_{u,t}$, the motion constraint of the pose graph can be mathematically formulated by quadratic forms of $\epsilon_{u,t}$ and $P_{u,t}$, as described in Equation (13). The registration edge is based on the semantic NDT which is described in Section 5.3.2. When mathematically defining the semantic registration $T(x_{t-1},p)$ as $h_{s}\left(x_{t-1},s_{t-1},s_{t},\Theta \right)$, the registration edge is expressed by Equation (14), similar to the motion edge.

(14) $$\begin{array}{c} x_{t}=h_{s}\left(x_{t-1},s_{t-1},s_{t},\Theta \right)+\epsilon_{s,t}\\ {\left(x_{t}-h_{s}\left(x_{t-1},s_{t-1},s_{t},\Theta \right)\right)}^{T}P_{s,t}^{-1}\left(x_{t}-h_{s}\left(x_{t-1},s_{t-1},s_{t},\Theta \right)\right) \end{array}$$

The covariance of the semantic NDT, $P_{s,t}$, is estimated based on a Hessian-matrix-based approach introduced in [43].

The optimized vehicle poses can be obtained by minimizing the cost function derived in the previous section. The cost function for Graph SLAM is obtained by the summation of the log-likelihood constraints in the graph, as represented in Equation (15).

(15) $$\begin{array}{c} J\left(x\right)=\sum_{t}\epsilon_{m}{\left(x_{t}\right)}^{T}P_{m,t}^{-1}\epsilon_{m}\left(x_{t}\right)+\sum_{t}\epsilon_{s}{\left(x_{t}\right)}^{T}P_{s,t}^{-1}\epsilon_{s}\left(x_{t}\right) \end{array}$$

(16) $$x^{*}=arg\min_{x}J(x)$$

In order to minimize the cost function, an open-source framework for a non-linear least square problem, g2o, is adopted [44]. The solver provides the optimized vehicle poses in the graph as described in Equation (16).

### 6.2. Probabilistic Point Cloud Accumulation

Based on the optimized vehicle poses and the segmented point clouds, the final step of the proposed semantic mapping framework is semantic map generation by probability-based point cloud accumulation. The basic of the point map generation is to accumulate all of the segmented point clouds transformed into the global frame using the optimized poses as time going. However, one point measured from different poses can indicate the different semantic information because of the uncertainty of the class classification, which causes ambiguity of the semantic map. In order to solve this problem, this paper proposes a probability-based point cloud accumulation to determine the semantic class of the points. As explained before, the probability of a point representing the true class *C* is defined as the conditional probability $p(C|C_{i})$, where $C_{i}$ is the predicted class from the semantic segmentation. With an assumption that the class probabilities of points from the different poses are independent, the probability of class *C* can be updated by multiplication $p_{t-1}\left(C|C_{i}\right)\cdot p_{t}\left(C|C_{j}\right)$, where $C_{i}$ and $C_{j}$ are the predicted classes of the point from the time t−1 and *t*, respectively. Consequently, after time has gone, the probability of the class $C_{k}$ is determined as modeled in Equation (17).

(17) $$\begin{array}{c} P\left(C_{k}\right)=\prod_{t\in W}^{}p_{t}\left(C_{k}|C_{pred,t,i}\right)\;,where\;k\in \forall C \end{array}$$

**Figure 10:**
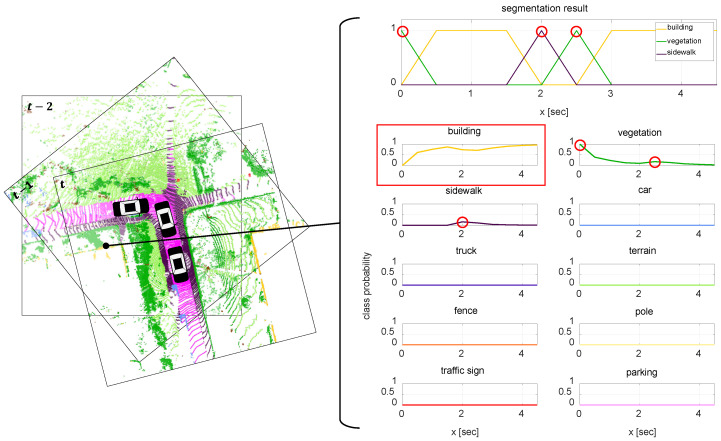
Class probability update. The probability of each point’s class is consistently updated until the point is out of the field of view of the sensor.

Here, $C_{pred,t,i}$ is a predicted class of *i*th point in time *t* and *W* is the time window which describes duration when the point *i* is measured. In the same manner, an arbitrary point has probabilities for all classes in the set C. As a result, the semantic class of the point can be determined as the class with the maximum probability as shown in Figure 10. In this figure, the class probability of a point is updated by the proposed probability model. Even though the point is classified as *vegetation* at time 0 s, 2.5 s and as *sidewalk* at time 2 s, the class is determined as *building* when the update is completed.

## 7. Experiment

The proposed semantic mapping framework was evaluated in both KITTI datasets and real driving environments. The experimental evaluation is aimed to support our main contributions that 1) Probability models for the uncertainty of the deep neural network-based semantic segmentation 2) Probability model-based semantic mapping. To do this, this section consists of four sub-sections. The Section 7.1 provides the information about experiment environments. The explanation of the SemanticKITTI and of the real driving environments including vehicle configuration are covered. Next, Section 7.2 presents the results of deep-neural network-based semantic segmentation. This part also provides the learning parameters as well as the segmentation results. The Section 7.3 describes the probability-based semantic registration. In this part, the prior knowledge from the SemanticKITTI is quantified as the proposed two probability models. Also, the optimized vehicle trajectory of the proposed framework is compared with other LiDAR SLAM methods using KITTI datasets and our driving data. The generated semantic map is presented in Section 7.4. The semantic map from the proposed framework reduces the semantic ambiguity from the segmentation uncertainty by the probabilistic update of the point’s semantic class.

### 7.1. Experiment Environments

**Figure 11:**
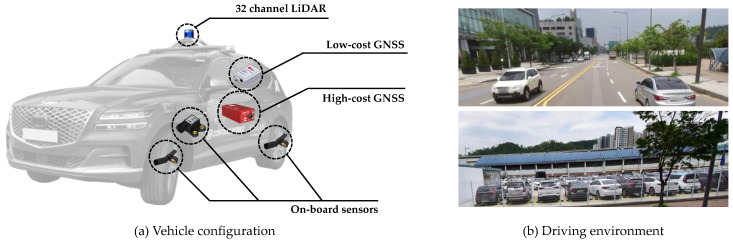
Experiment environment for the outdoor evaluation. (**a**) Test vehicle *A1* equipped with 32-channel LiDAR, on-board sensors, low-cost GNSS, and high-cost GNSS. (**b**) Test site including urban roads and parking lot.

The SemanticKITTI provides the point-wise annotations of KITTI Odometry Benchmark. It contains over 20,000 point cloud data from Velodyne HDL-64E LiDAR with a rate of 10 Hz and those data were collected from 22 different driving environments such as urban roads, highways, and countryside. Also, the dataset annotates point cloud into 25 classes including the static objects such as building, pole, and parked car as well as the dynamic objects like moving car, person, and so on. It also provides ground truth vehicle poses from high-cost GNSS/INS. The labeled point clouds are used for training the semantic segmentation networks and for the prior knowledge definition, and the unlabeled point clouds in the KITTI datasets are used for the evaluation of the proposed algorithm. The evaluation in real driving environments is conducted using the autonomous car *A1* of Hanyang University, as shown in Figure 11a. The test vehicle is equipped with 32-channel LiDAR, on-board sensors, and a low-cost GNSS sensor for the localization. The RTK (Real-Time Kinematic) GNSS was used for a high-cost GNSS for a reference sensor for the evaluation. The outdoor evaluation dataset includes both a highly dynamic urban scenario with a lot of moving objects and a parking lot with a lot of parked vehicles, which is challenging for the SLAM problem as shown in Figure 11b. The proposed algorithm is run on Intel i5-4690 CPU @3.5GHz with 16GB RAM. The GPU for semantic segmentation is performed on Nvidia RTX 2080 GPU with 8GB RAM. The semantic segmentation algorithms are run within 100ms which is shorter than the LiDAR data frequency.

### 7.2. Semantic Segmentation

**Figure 12:**
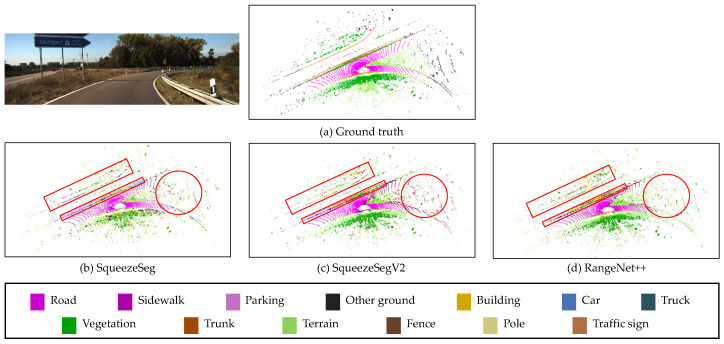
Semantic segmentation results of three different deep neural networks. (**a**) Ground truth from SemanticKITTI. (**b**) Segmentation result of SqueezeSeg. (**c**) Segmentation result of SqueezeSegV2. (**d**) Segmentation result of RangeNet++.

The three semantic segmentation models (SqueezeSeg, SqueezeSegV2, RangeNet++) are trained by the SemanticKITTI dataset. Among the sequences 00 to 10, sequence 08 is used for validation and the remaining sequences are used for the training, as the same with [8]. The training is performed on Nvidia RTX 2080ti GPU. For the hyper-parameters for the training, the maximum epoch is 150, the initial learning rate is 0.001, and the learning rate decay is 0.99 per 1 epoch. The size of the projected image is (2046, 64) for laser scan data. These hyper-parameters were evaluated by the papers which proposed these semantic segmentation models [8,25,26]. The studies used these parameters and provided the successful segmentation results. The segmentation results of three segmentation models for sequence 01 (Highway scene) are described in Figure 12, in which the semantic class information is represented by color. As shown in the red markers in this figure, the algorithms cannot always provide true semantic information for the surrounding objects, which causes the uncertainty of the semantic information. Furthermore, the semantic segmentation algorithms are executed within 100 milliseconds (the data duration of the LiDAR point clouds) as shown in Table 1.

### 7.3. Uncertainty Probability-Based Registration

In this part, the uncertainty of the semantic segmentation is modeled as probabilities using the prior knowledge. Again, there are two uncertainties covered in this paper: movability and classification performance. The movability is modeled by the intra-class probability and the classification performance is modeled by the inter-class probability. The modeled probabilities are used for the point cloud registration in order to reflect the uncertainty of the semantic segmentation algorithms.

#### 7.3.1. Prior Knowledge-Based Class Probability

**Figure 13:**
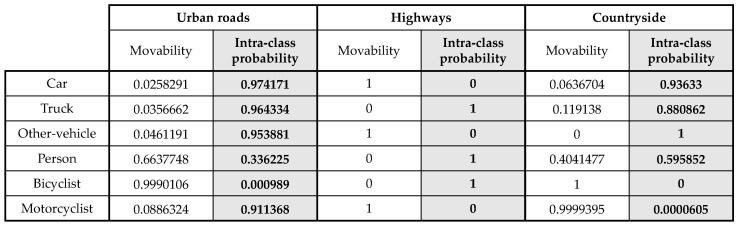
Intra-class probability from the movability by analysis of the SemanticKITTI.

The intra-class probability is directly derived from the movability which is analyzed with the SemanticKITTI dataset. Because the dataset provides the independent labels for the moving objects, the movability of each sequence can be obtained by Equation (2). However, the movability of the objects is quite different according to driving environments. For example, on the highway the movability of car is high, however, in the parking lot, the movability is relatively low. In order to consider the environmental dependency, all of the points in the SemanticKITTI dataset are categorized as scene-by-scene by three representative environments; urban roads, highways, and countryside. Consequently, three intra-class probability tables are generated for each class as shown in Figure 13. The number of points of each environment is about 1.7 billion, 0.1 billion, and 0.9 billion for urban roads, highways, and countryside, respectively. As expected, in the highways, all of the points labeled by car, motorcyclist, and other-vehicle are moving. The reason why the movabilities of truck, person, and bicyclist are zero is that there is no point labeled by them in the SemanticKITTI. For the urban roads and countryside, most of the car-like objects are not labeled as *moving objects* because they are parked. So, the intra-class probabilities for them are high, which means that the points can be highly used for the registration.

**Figure 14:**
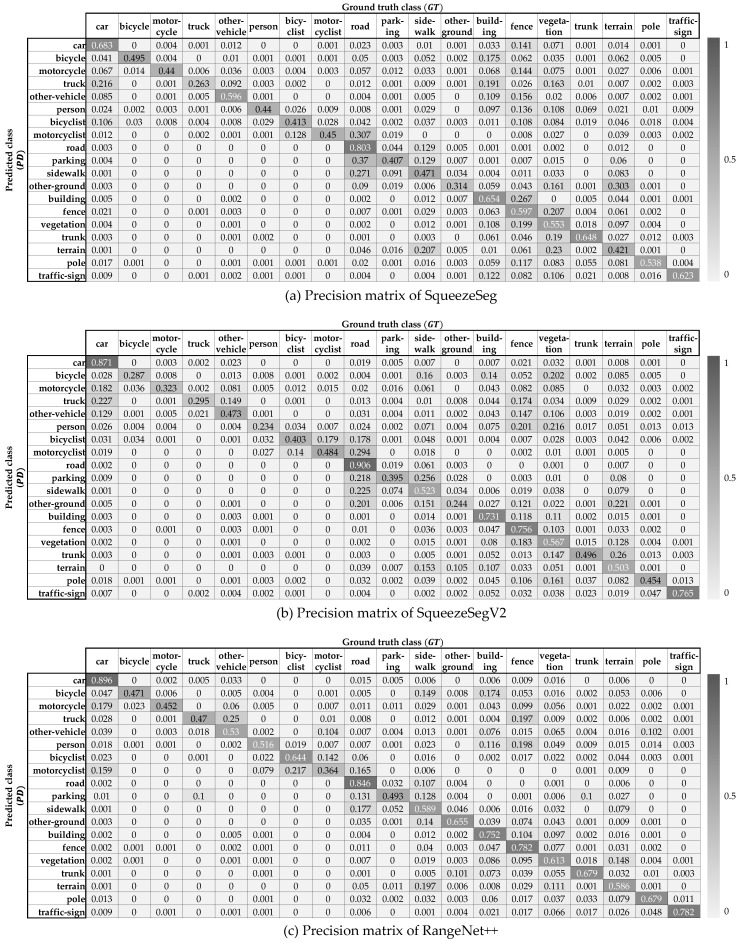
Precision matrix of the semantic segmentation algorithms.

**Figure 15:**
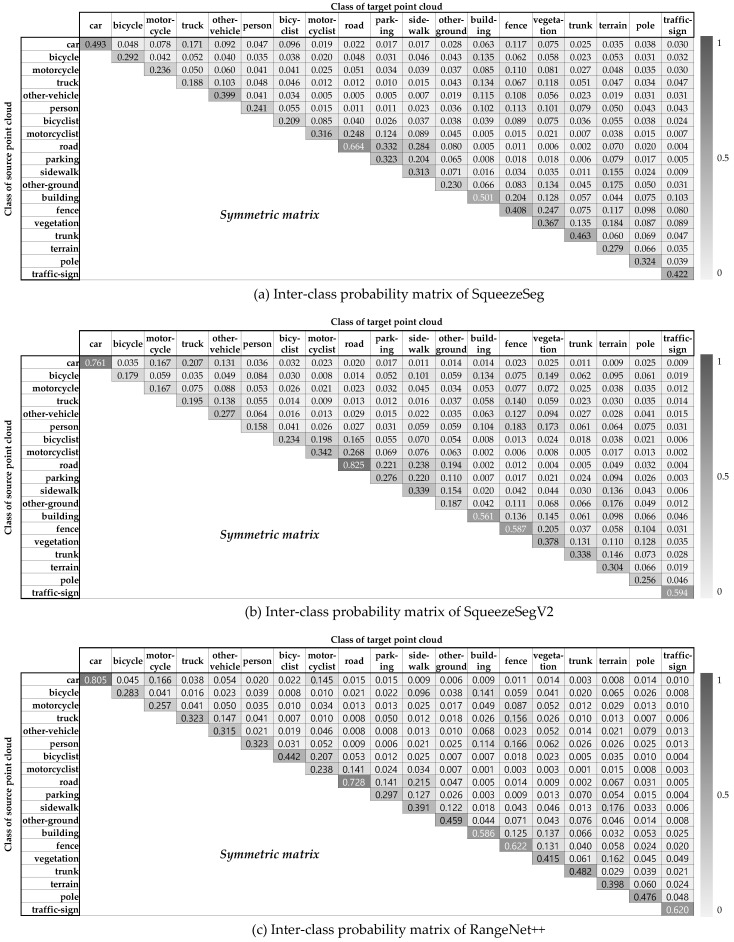
Inter-class probability matrix of the semantic segmentation algorithms.

The inter-class probability is derived from the confusion matrix which represents the classification performance. In order to cover the general environments, three semantic segmentation algorithms are trained and tested for all the sequences in the SemanticKITTI. When the algorithms are tested in sequence 00, they are trained by sequence 01 to 10, and so does the rest. This training process is only for the inter-class probability, which is independent with the Section 7.2. As a result, the total confusion matrix for one semantic segmentation algorithm is obtained by summation of the confusion matrices for all the sequences. As explained in Equation (3), the conditional probability $p\left(C_{t}|C_{i,j}\right)=\frac{n(PD_{C_{i,j}}\cap GT_{C_{t}})}{n(PD_{C_{i,j}})}$ called as *precision* is used for the intra-class probability. The precision matrices from the confusion matrices of three deep neural networks are shown in Figure 14. As a result, the inter-class probability for all the pairs can be calculated from Equation (3), which is described in Figure 15. This probability matrix is used for the weight factor of the semantic NDT as described in Equation (11). When the classes of two matching points segmented by the algorithms are the same(values of the principal diagonal of the matrix), the probability is relatively high, however, they are not 1. On the other hand, even if the matching points are segmented as different classes, they are used for the semantic registration with their inter-class probability values because there is classification uncertainty of the semantic segmentation algorithms.

#### 7.3.2. Semantic Registration-Based Pose Optimization

**Figure 16:**
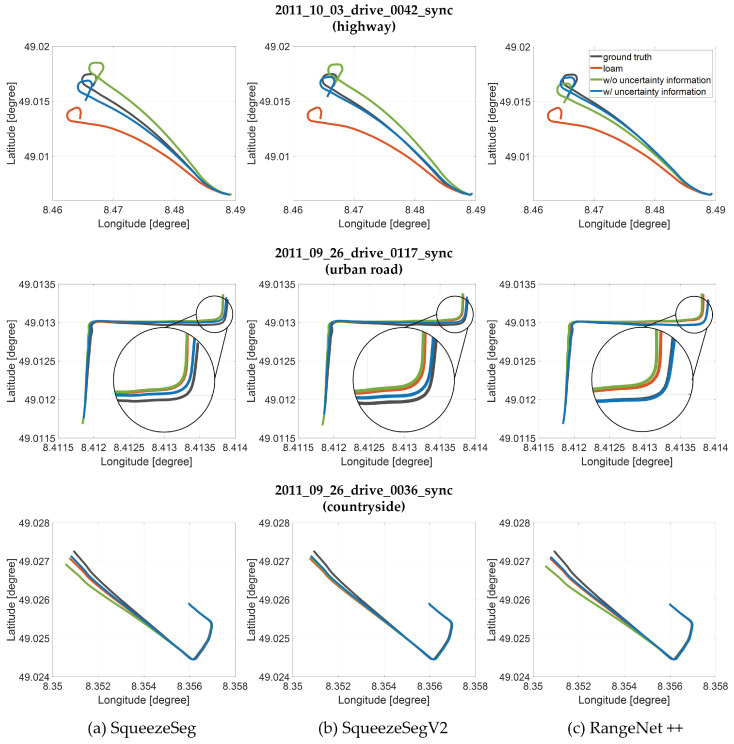
Evaluation of the proposed algorithm with KITTI datasets with three semantic segmentation algorithms (trajectory evaluation).

**Figure 17:**
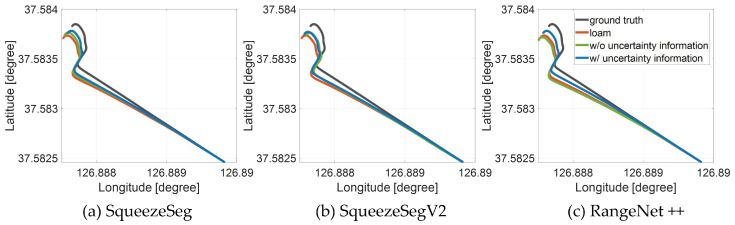
Evaluation of the proposed algorithm in outdoor environment with three semantic segmentation algorithms using RTK-GNSS as ground truth trajectory (trajectory evaluation).

This section shows the mapping trajectories of the proposed mapping algorithm using KITTI datasets and our outdoor dataset. The proposed mapping trajectory is from the semantic registration-based pose optimization based on the two probability models (Figure 13 and Figure 15). The mapping trajectory is compared with two types of previous approaches; Basic LiDAR SLAM without semantic information, Semantic mapping without the explicit model of uncertainty. More specifically, LOAM [16] is adopted for the basic LiDAR SLAM, and SuMa++ [7] is selected for the comparison with the semantic mapping only when the RangeNet++ is used for the semantic segmentation algorithm. Figure 16 shows the trajectory evaluation results using KITTI datasets. Three raw datasets in the KITTI are used for the evaluation in order to verify the proposed semantic mapping in the overall driving environments. These datasets were not used both for the training of semantic segmentation algorithm and for the prior knowledge definition. The raw indicates the same datasets and the column indicates the semantic segmentation algorithm. For all cases, the trajectory from the proposed algorithm (blue line) is closest to the ground truth trajectory (gray line). The outdoor evaluation results with our driving data are shown in Figure 17. In the same manner, it is verified that the proposed mapping algorithm generates a consistent trajectory compared with previous approaches.

Because the trajectory evaluation can be insufficient due to the drift effects, the quantitative results of the proposed framework in terms of translation and rotation error are also shown in Table 2. The translation and rotation errors are obtained by comparing the transformation relationship between the ground truth (RTK-GNSS for outdoor evaluation) and the registration results. As shown in this table, LOAM provides a lower maximum rotation error in the urban road and lower RMS translation error in the countrysides when the SqueezeSegV2 is used for the semantic segmentation algorithm. It is a reasonable result because there are a lot of buildings in the datasets and LOAM uses a feature-based registration method. Moreover, in the highway dataset, SuMa++ shows better performance in the RMS translation error and the maximum rotation error, because SuMa++ aimed at the reliable mapping in the highways including moving objects. However, the proposed algorithm provides lower transformation errors in the other scenarios including our driving data with dynamic urban roads and parking lot. In summary, the proposed algorithm is evaluated in the overall driving environments with various semantic segmentation algorithms.

### 7.4. Probabilistic Semantic Mapping

**Figure 18:**
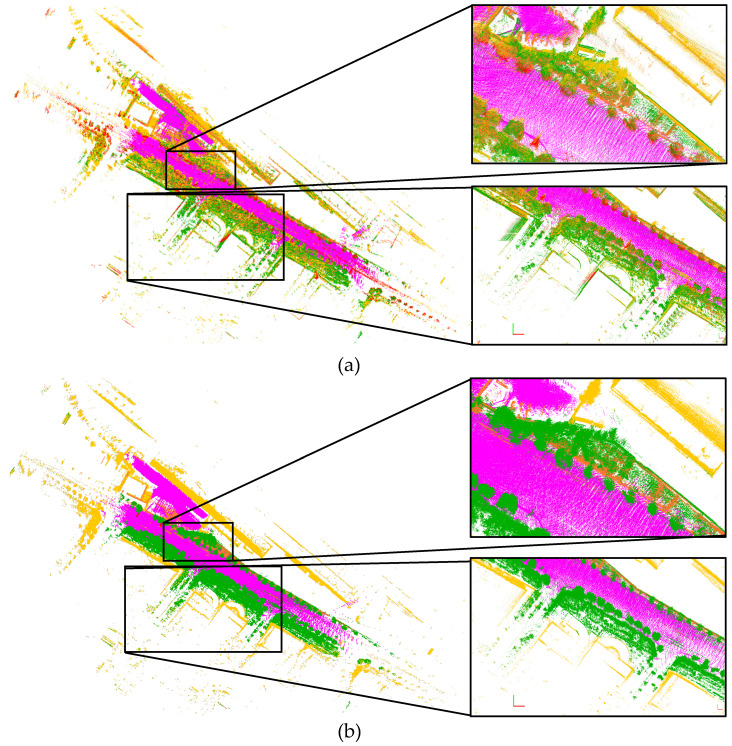
Semantic map generated by the proposed semantic mapping. (**a**) semantic map without probabilistic accumulation. (**b**) semantic map from the proposed probabilistic accumulation.

The final experiment result is for the probabilistic semantic mapping. The semantic map is generated by the accumulation of the segmented point cloud based on the optimized mapping trajectory. The evaluation result about the generated semantic map is shown in Figure 18. The semantic map is built using the trajectory from the proposed algorithm with our driving data, and RangeNet++ is used for semantic segmentation. As shown in Figure 18a, one object contains multiple semantic classes, which causes the ambiguity of the semantic map. This is because the multiple scans from different poses indicate the different semantic classes due to the classification uncertainty. In the proposed method, the precision matrix (Figure 14) is used for the probabilistic update of the semantic class in order to reflect the classification uncertainty. The class probabilities for all classes are consistently updated by the proposed update model (Equation (17)), consequently the semantic ambiguity is reduced as shown in Figure 18b.

## 8. Conclusions

This paper proposed semantic segmentation-based LiDAR mapping reflecting the uncertainty of the deep neural network-based segmentation algorithms as probability models. The state-of-the-art semantic segmentation algorithms were used for the point cloud segmentation. The uncertainty of the segmentation was modeled by two data-based probability models; inter-class and intra-class probability. These probabilities were utilized for the point cloud registration in the proposed semantic registration. Based on the proposed registration, the mapping trajectory was optimized by Graph SLAM. The final semantic map was constructed by the proposed probabilistic accumulation of the segmented point clouds. The main advantages of the proposed mapping framework are summarized as follows:

1. The uncertainty of the semantic segmentation algorithms was modeled by data-driven approaches. The uncertainty about the movability of a class was quantified by the intra-class probability, and the uncertainty about classification performance was modeled by the inter-class probability. These probability models were derived from the labeled point clouds from various environments (SemanticKITTI), which means that the proposed probability models can be applied to the overall driving environments.
2. The semantic registration was proposed to apply the probability models to point cloud registration. Instead of using the semantic classes for the registration directly, the proposed registration combined the NDT registration and uncertainty probabilities. The intra-class probability was used to generate the multi-modal distribution and the inter-class probability played a role as the weight factor for the NDT optimization. Based on the semantic registration, the mapping trajectory was optimized by Graph SLAM with additional motion information.
3. The probabilistic semantic map building was proposed to reflect the uncertainty about the classification performance. In practice, because the multiple scans can indicate different classes for one point, the semantic ambiguity exists in the semantic map. In order to avoid ambiguity, this paper proposed the class probability update method using the precision matrix which represents the classification performance of the semantic segmentation algorithms. The class probability of points was consistently updated by the proposed method until the points were out of field-of-view.
4. The proposed mapping framework was evaluated with both KITTI datasets and outdoor driving dataset from our vehicle. First of all, the intra-class and inter-class probabilities were quantified as class-by-class tables. Next, the mapping trajectory of the proposed framework was evaluated by comparing it with representative LiDAR SLAM methods (LOAM, SuMa++). The evaluation datasets included the overall driving environments, and the proposed algorithm provided reliable trajectories. Furthermore, the transformation error of the mapping trajectory was compared with the previous algorithms. In specific environments, the previous algorithms showed better performance, however, the proposed algorithm provided lower errors in overall cases. Lastly, the generated semantic map was also verified. With the proposed probability-based mapping framework, the semantic ambiguity of the semantic map was reduced compared with the previous mapping algorithm.

Even though the proposed framework provides a reliable semantic map, there could still be semantic ambiguity due to the classification performance of the semantic segmentation algorithm. When the semantic segmentation repeatedly provided wrong semantic information for a point, the generated semantic map can indicate the wrong class of the point. In order to compensate for the problem, the authors plan to apply offline semantic segmentation to the generated semantic map. By applying the recursive segmentation, the more reliable semantic information is expected to be obtained even if the proposed semantic map contains the semantic ambiguity.

## Figures and Tables

**Table 1:** Mean and maximum processing time for the semantic segmentation algorithms.

Segmentation Algorithm	Processing Time [sec]	
	Mean	Max.
SqueezeSeg	0.076512	0.0844686
SqueezeSegV2	0.083942	0.0905341
RangeNet++	0.092457	0.0966730

**Table 2:** Translation and rotation error of the proposed semantic mapping algorithm. Bold numbers indicate best performance in terms of the errors.

Approach	Semantic Segmentation Method	Evaluation Sets							
		2011_10_03_drive_0042_sync (highway)		2011_09_26_drive_0117_sync (urban road)		2011_09_26_drive_0036_sync (countryside)		Our Driving Data (urban + parking lot)	
		Translation error [m] (rms/max)	Rotation error [deg] (rms/max)	Translation error [m] (rms/max)	Rotation error [deg] (rms/max)	Translation error [m] (rms/max)	Rotation error [deg] (rms/max)	Translation error [m] (rms/max)	Rotation error [deg] (rms/max)
LOAM	SqueezeSeg	0.1064/0.8304	0.3458/2.3848	0.0558/0.2697	0.1462/0.6002	0.0421/0.1416	0.1951/1.5459	0.2255/0.9948	0.2195/1.4098
	SqueezeSegV2	0.1064/0.8304	0.3458/2.3848	0.0558/0.2697	0.1462/**0.6002**	**0.0421**/0.1416	0.1951/1.5459	0.2255/0.9948	0.2195/1.4098
	RangeNet ++	0.1064/0.8304	0.3458/2.3848	0.0558/0.2697	0.1462/0.6002	0.0421/0.1416	0.1951/1.5459	0.2255/0.9948	0.2195/1.4098
Without uncertainty information	SqueezeSeg	0.0669/0.2885	0.1243/1.8346	0.0517/0.2869	0.1262/0.6624	0.0466/0.1523	0.2062/1.2514	0.2521/1.1344	0.2763/1.7189
	SqueezeSegV2	0.0721/0.4030	0.1779/1.3084	0.0514/0.2734	0.1384/0.7361	0.0450/0.1452	0.1995/2.0729	0.1940/1.1609	0.2795/1.7201
	RangeNet ++	**0.0629**/0.3412	0.1529/**1.0028**	0.0610/**0.2472**	0.1502/0.6207	0.0447/0.1349	0.1638/1.2727	0.1767/0.6582	0.2053/1.0387
With uncertainty information (Ours)	SqueezeSeg	**0.0666/0.2390**	**0.1123/1.0785**	**0.0481/0.2312**	**0.1230/0.5392**	**0.0382/0.1350**	**0.1564/0.9883**	**0.0832/0.4665**	**0.1779/0.6186**
	SqueezeSegV2	**0.0592/0.3214**	**0.1425/1.0463**	**0.0460/0.2632**	**0.1200**/0.6017	0.0444/**0.1373**	**0.1229/0.7499**	**0.1268/0.8664**	**0.1372/0.7690**
	RangeNet ++	0.0729/**0.2632**	**0.1501**/1.3290	**0.0547**/0.2935	**0.1277/0.4743**	**0.0378/0.1342**	**0.1211/0.6879**	**0.0602/0.3695**	**0.1461/0.9659**
